# Supplementary material for: Transcriptome Analysis of the Necrotrophic Pathogen Alternaria brassicae Reveals Insights into Its Pathogenesis in Brassica juncea
Source: Microbiol Spectr. 2023 Mar 13;11(2):e02939-22. doi: 10.1128/spectrum.02939-22 (PMC10100672; doi:10.1128/spectrum.02939-22)
Supplement: Supplemental file 1 — Supplemental material. Download spectrum.02939-22-s0001.pdf, PDF file, 1.3 MB [file spectrum.02939-22-s0001.pdf]

Transcriptome analysis of the necrotrophic pathogen *Alternaria brassicae* reveals insights into its pathogenesis in *Brassica juncea*

Supplemental Figures S1-4.

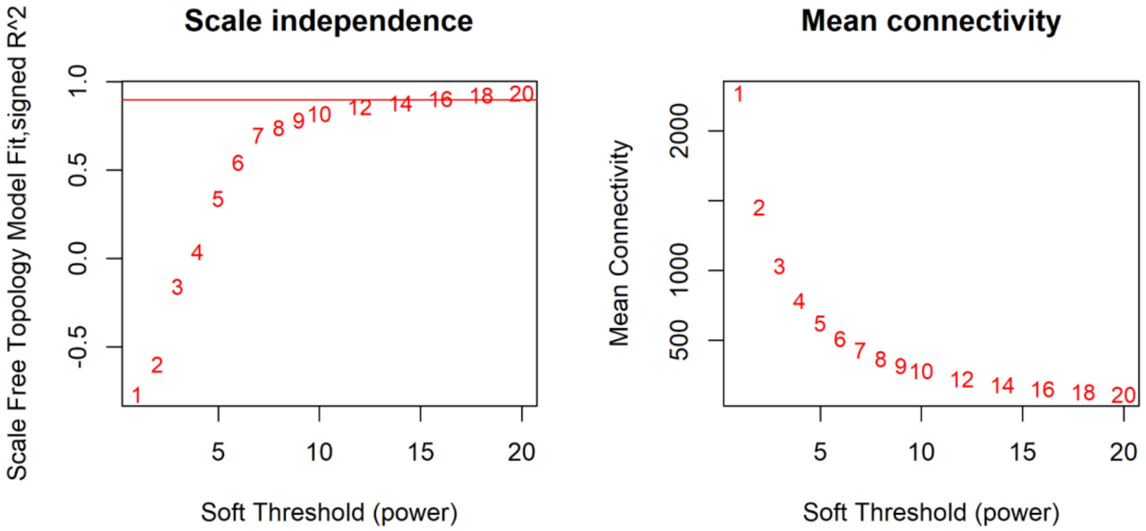

Figure S1: Softpower threshold estimation in WGCNA

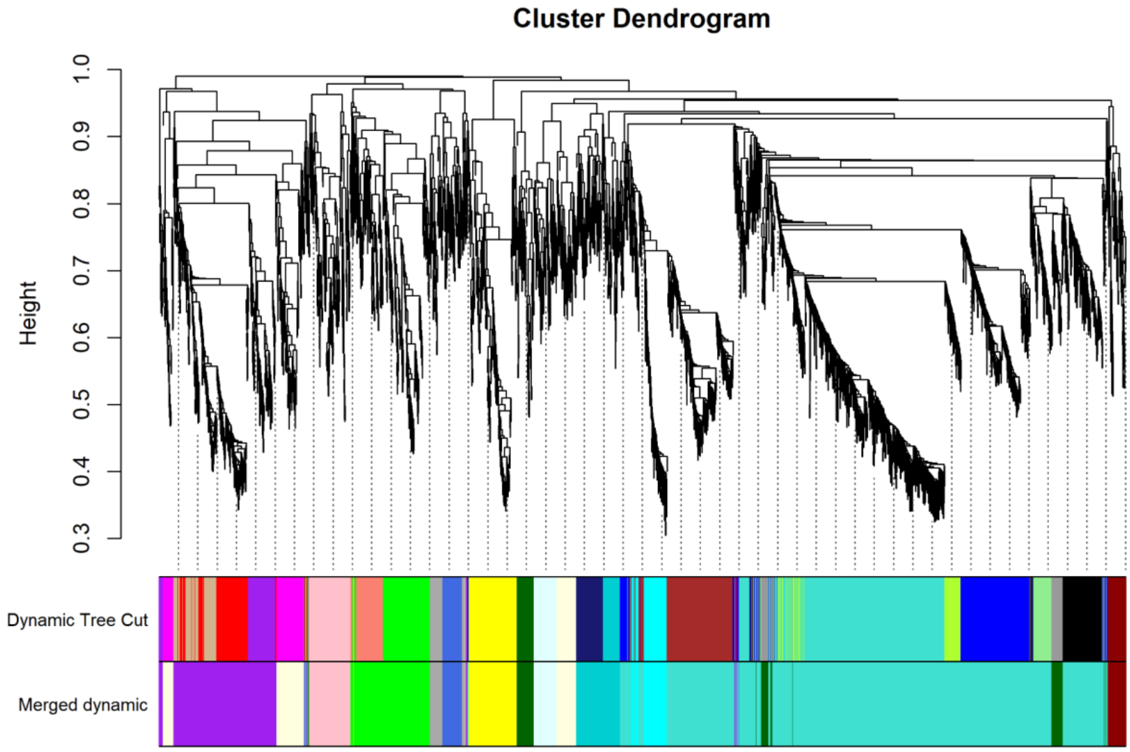

Figure S2: DEGs and their module membership before and after DynamicTree Cut

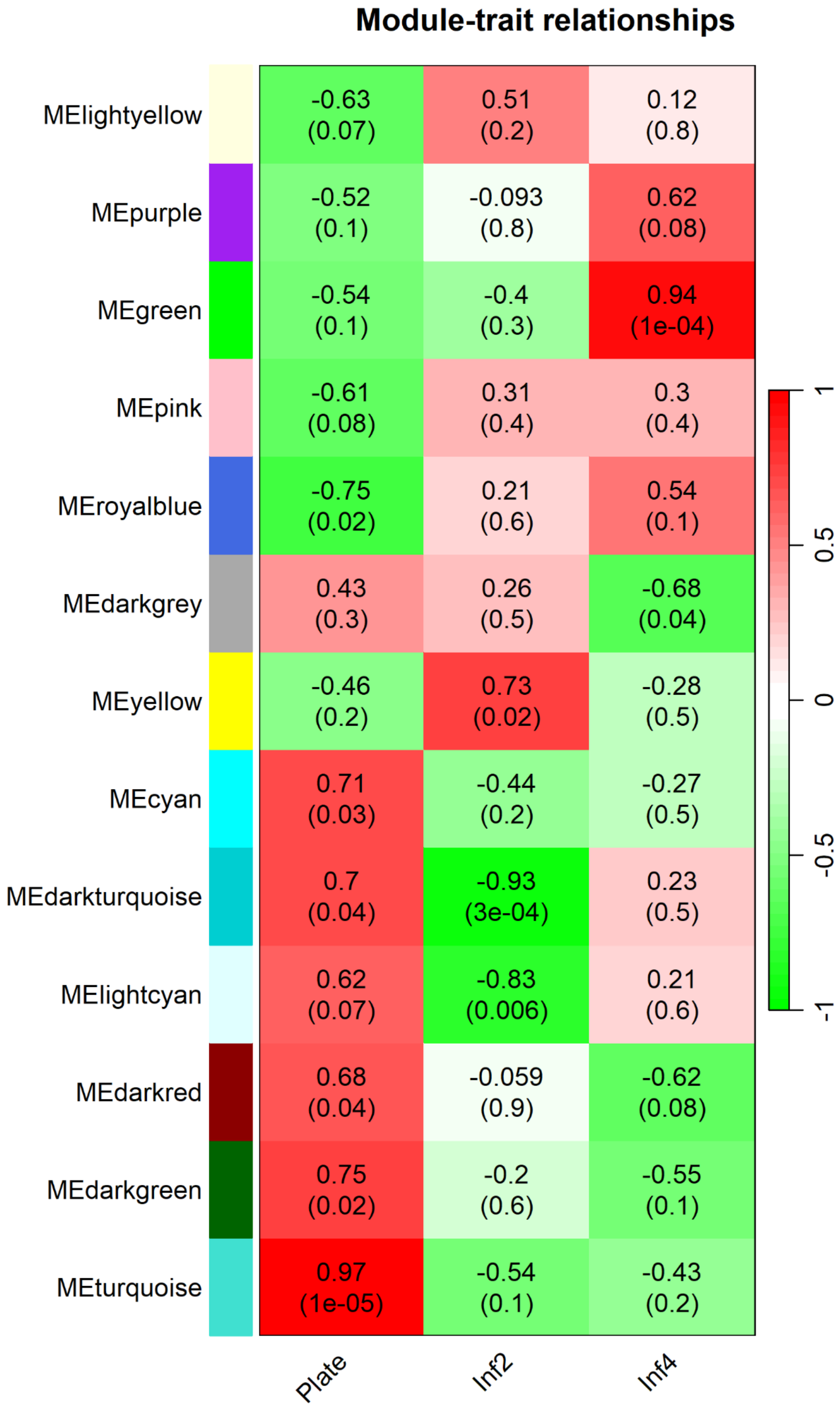

Figure S3: Module-trait correlation analysis in WGCNA. Each module was correlated to the sample type (*in vitro*, 2 dpi, 4 dpi).

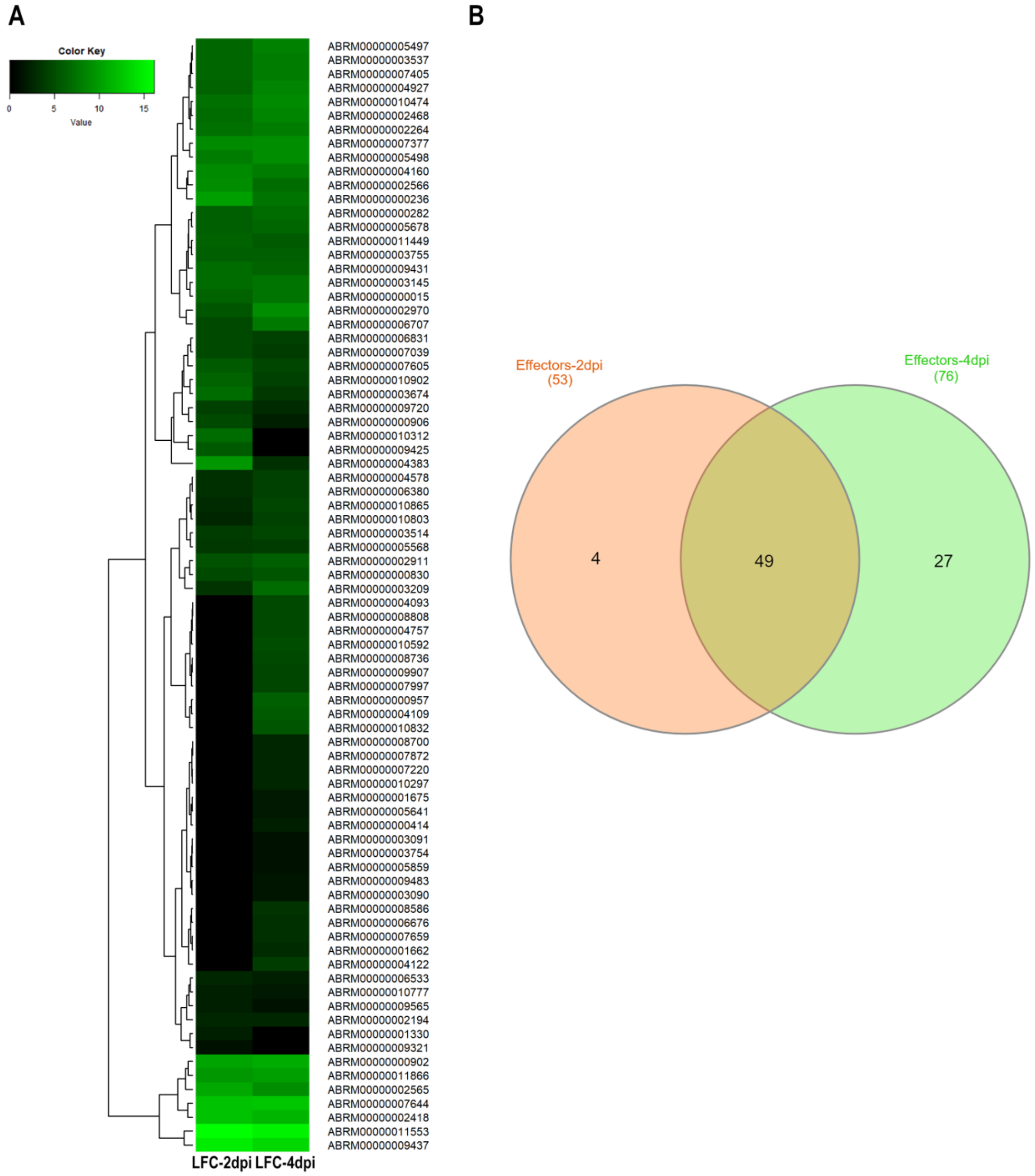

Figure S4: Gene expression profile of effectors of *A. brassicae*. (A) Heatmap depicting the $\log_2$ FoldChange values of 80 effectors at 2 and 4 dpi. (B) Venn diagram representing the overlap between the effectors significantly upregulated at 2 and 4 dpi.
